# High-Resolution MR Imaging of the Human Brainstem *In vivo* at 7 Tesla

**DOI:** 10.3389/fnhum.2013.00710

**Published:** 2013-10-29

**Authors:** Andreas Deistung, Andreas Schäfer, Ferdinand Schweser, Uta Biedermann, Daniel Güllmar, Robert Trampel, Robert Turner, Jürgen R. Reichenbach

**Affiliations:** ^1^Medical Physics Group, Institute of Diagnostic and Interventional Radiology I, Center of Radiology, Jena University Hospital – Friedrich Schiller University Jena, Jena, Germany; ^2^Department of Neurophysics, Max Planck Institute for Human Cognitive and Brain Sciences, Leipzig, Germany; ^3^Institute of Anatomy I, Jena University Hospital – Friedrich Schiller University Jena, Jena, Germany

**Keywords:** brainstem, quantitative susceptibility mapping, effective transverse relaxation, longitudinal relaxation, diffusion tensor imaging, track-density imaging, brain, anatomy

## Abstract

The human brainstem, which comprises a multitude of axonal nerve fibers and nuclei, plays an important functional role in the human brain. Depicting its anatomy non-invasively with high spatial resolution may thus in turn help to better relate normal and pathological anatomical variations to medical conditions as well as neurological and peripheral functions. We explored the potential of high-resolution magnetic resonance imaging (MRI) at 7 T for depicting the intricate anatomy of the human brainstem *in vivo* by acquiring and generating images with multiple contrasts: *T*_2_-weighted images, quantitative maps of longitudinal relaxation rate (*R*_1_ maps) and effective transverse relaxation rate ($R_{2}^{*}$ maps), magnetic susceptibility maps, and direction-encoded track-density images. Images and quantitative maps were compared with histological stains and anatomical atlases to identify nerve nuclei and nerve fibers. Among the investigated contrasts, susceptibility maps displayed the largest number of brainstem structures. Contrary to *R*_1_ maps and *T*_2_-weighted images, which showed rather homogeneous contrast, $R_{2}^{*}$ maps, magnetic susceptibility maps, and track-density images clearly displayed a multitude of smaller and larger fiber bundles. Several brainstem nuclei were identifiable in sections covering the pons and medulla oblongata, including the spinal trigeminal nucleus and the reticulotegmental nucleus on magnetic susceptibility maps as well as the inferior olive on *R*_1_, $R_{2}^{*}$, and susceptibility maps. The substantia nigra and red nuclei were visible in all contrasts. In conclusion, high-resolution, multi-contrast MR imaging at 7 T is a versatile tool to non-invasively assess the individual anatomy and tissue composition of the human brainstem.

## Introduction

The brainstem is the primary relay center for afferent and efferent connections between the cerebral cortex, the cerebellum, and the spinal cord (Sutin and Carpenter, 1976). It plays an important role in the regulation of vital functions (e.g., respiratory function, cardio-vascular function, nausea) and coordinates motor control signals sent from the brain to the body. Anatomically, the human brainstem consists of three connected parts, medulla oblongata, pons, and midbrain, and is composed of a multitude of axonal nerve fibers as well as cranial and non-cranial nerve nuclei. Due to the spatial concentration of important neural structures in this relatively small brain region, pathological variations or lesions in the brainstem can introduce severe and multiple neurological effects (Donaldson et al., 2006). The brainstem is also known to be involved in a number of degenerative diseases, such as Alzheimer’s disease, Parkinson’s disease, and Huntington’s disease (Urban and Caplan, 2011). Hence, detailed non-invasive depiction of its morphology and anatomy may help to better relate normal and pathological anatomical variations to medical conditions as well as neurological and peripheral functions.

Magnetic resonance imaging (MRI) at ultrahigh magnetic field strength (*B*_0_ ≥ 7 T) is a powerful means to assess non-invasively normal and abnormal brain tissue with high spatial resolution (Li et al., 2006; Duyn et al., 2007; Deistung et al., 2008; Trampel et al., 2011; Moser et al., 2012; Turner, 2012, 2013; Eichner et al., 2013; Marques and Gruetter, 2013). MRI provides a variety of qualitative and quantitative tissue contrasts, mainly reflecting nuclear relaxation (*T*_1_, *T*_2_, and $T_{2}^{*}$, or equivalently *R*_1_, R_2_, and $R_{2}^{*}$), diffusion, and magnetic susceptibility. In contrast-sensitized MR images (e.g., *T*_1_-weighted, *T*_2_-weighted, or $T_{2}^{*}$-weighted images), however, the measured signal is an intricate function of proton density, longitudinal (*T*_1_) and transverse relaxation (*T*_2_) and depends on the applied MRI sequence parameters. Thus, it may be difficult to interpret the underlying tissue composition and structure based on the intensity of these images. Quantitative MRI techniques, on the other hand, provide information that is intrinsically more tissue specific and consequently less dependent on the chosen scan parameters. Hence, quantification of different physical tissue properties by using quantitative MRI techniques also increases intra- and inter-individual comparability and enables objective measurements of disease related brain changes (Tofts, 2003).

The most common approach of quantitative MRI is relaxometry, i.e., mapping of the longitudinal (*R*_1_) and/or transverse (R_2_, $R_{2}^{\prime }$, $R_{2}^{*}$) relaxation rates. In the brain these relaxation rates are influenced predominantly by both myelin (Koenig, 1991; Bender and Klose, 2010; Langkammer et al., 2012a; Lee et al., 2012) and tissue iron (Ogg and Steen, 1998; Langkammer et al., 2010). An additional, recently introduced novel quantitative MR method that is unique in its sensitivity to both tissue constituents is quantitative susceptibility mapping (QSM) (Schweser et al., 2011; Reichenbach, 2012). QSM is a post-processing method that uses the phase signal of complex-valued gradient (recalled) echo (GRE) data to produce maps of tissue magnetic susceptibility. Quantification of brain tissue iron content (Wharton et al., 2010; Schweser et al., 2011; Bilgic et al., 2012; Langkammer et al., 2012b; Zheng et al., 2013) and blood oxygenation *in vivo* (Haacke et al., 2010) as well as assessment of brain myelination (Liu et al., 2011; Li et al., 2012a,b) has recently been reported by applying QSM. Excellent anatomical delineation of cortical and deep gray matter structures and substructures has been demonstrated with QSM (Schweser et al., 2011, 2012; Deistung et al., 2013a), particularly in concert with relaxation rate mapping (Sigalovsky et al., 2006; Fukunaga et al., 2010; Geyer et al., 2011; Lebel et al., 2012; Deistung et al., 2013a), thus rendering this combined approach highly promising for improved delineation of the anatomical structure of the brainstem.

Nerve fibers and their complex network are usually imaged and analyzed by exploiting the translational molecular diffusion of protons. So far, most studies have applied diffusion tensor imaging (DTI) (Basser and Pierpaoli, 1996) for anatomical imaging of the brainstem (Stieltjes et al., 2001; Nagae-Poetscher et al., 2004; Salamon et al., 2005). DTI, however, is commonly limited by the relatively coarse spatial resolution available, which makes detailed analysis of smaller fiber tracts difficult, and often fails in regions of crossing fibers (Jones et al., 2013). One recently suggested approach to overcome this limitation is track-density imaging (TDI), which combines high angular resolution diffusion imaging (HARDI) with fiber tracking information to delineate structures beyond the image resolution of the acquired diffusion-weighted data (Calamante et al., 2010). TDI has been already applied successfully to delineate fiber pathways in the cerebrum and cerebellum, and the substructure of the thalamus (Calamante et al., 2010, 2013).

The present study explores the potential of these high-resolution MRI approaches to resolve non-invasively the intricate anatomy of the human brainstem at 7 T *in vivo*. To this end, *T*_2_-weighted images, quantitative *R*_1_, $R_{2}^{*}$, and magnetic susceptibility maps as well as track-density images were compared with respect to their ability of depicting nerve nuclei and nerve fibers. Furthermore, *R*_1_, $R_{2}^{*}$, and magnetic susceptibility values are presented for selected anatomical regions. It is shown that the combination of multiple image contrasts provides a distinctly improved portrayal of the morphology of the brainstem.

## Materials and Methods

### Data acquisition

The study was approved by the internal Institutional Review Board of the Max Planck Institute in Leipzig and written informed consent was obtained from all participating subjects. A total of six healthy volunteers (four male and two female; 27.7 ± 3 years) were examined on a 7 T human whole body MRI system (Siemens Healthcare, Erlangen, Germany) using a 24-channel head array coil (NOVA Medical Inc., Wilmington, MA, USA). Two dielectric pads containing deuterated water (99%, Sigma Aldrich GmbH, Germany) enriched with calcium titanate (Alfa Aesar GmbH and Co KG, Karlsruhe, Germany) were placed on the left and right side of the subjects’ head to increase both field strength and homogeneity of the transmit radio frequency field, $B_{1}^{+}$, in the area of the brainstem (Teeuwisse et al., 2012).

Coronal *T*_2_-weighted images were acquired using a two-dimensional gradient-echo and spin-echo (GRASE) sequence (Feinberg and Oshio, 1991; Oshio and Feinberg, 1991) with echo time (TE) = 35 ms, repetition time (TR) = 10,000 ms, bandwidth (BW) = 343 Hz/px, acquisition matrix = 384 × 384 and in-plane resolution of 0.53 mm × 0.53 mm. Thirty-five images were collected with a slice thickness of 0.6 mm and a gap between two adjacent slices of 0.3 mm. For each TR interval there were three 180°-refocusing pulses, and between each 180°-pulse there were three gradient recalled echoes, resulting in an effective echo train length of nine and an acquisition time (TA) of 7:22 min:s. The GRASE sequence was applied to account for the high specific absorption rate (SAR) that substantially restricts *T*_2_-weighted imaging with turbo-spin-echo sequences at ultra-high magnetic fields.

High-resolution *R*_1_-mapping was performed based on the data acquired with the MP2RAGE sequence (Marques et al., 2010), a magnetization-prepared rapid gradient-echo (MP-RAGE) sequence with two different inversion times (TI). The acquisition parameters of the coronal MP2RAGE scan included TI_1_ = 900 ms, TI_2_ = 2750 ms, TE = 3.7 ms, TR = 5000 ms, BW = 240 Hz/px, acquisition matrix = 320 × 260 × 240, voxel size = 0.6 mm × 0.6 mm × 0.6 mm, and TA = 17:40 min:s. Magnetization preparation was achieved with a tailored radiofrequency pulse to take into account the heterogeneity of the radiofrequency transmit field (Hurley et al., 2010).

Coronal, multi-echo, three-dimensional gradient-echo imaging was carried out to compute quantitative $R_{2}^{*}$ and susceptibility maps. To this end, three echoes with monopolar readout were recorded (TE_1_ = 11 ms, TE_2_ = 21 ms, and TE_3_ = 31 ms), TR = 43 ms, flip angle (FA) = 12.5°, BW = 149 Hz/px, acquisition matrix = 448 × 364 × 104, and voxel size = 0.43 mm × 0.43 mm × 0.43 mm. Data were collected with 75% and 87.5% partial Fourier along phase and slice encoding direction, respectively, resulting in an acquisition time of 17:48 min:s. Choosing a readout BW of 149 Hz/px yielded single-echo images with a rather high signal-to-noise ratio (SNR), however, at the expense of a rather long inter-echo distance. The signal decay was sampled with three echoes only to keep acquisition time below 20 min.

Finally, diffusion-weighted imaging (DWI) data were acquired in sagittal orientation with two-dimensional, single-shot, spin-echo echo-planar imaging (EPI), 60 diffusion encoding directions each with a b-value of 1000 s/mm^2^ and seven volumes with a *b*-value of 50 s/mm^2^, TE = 64 ms, TR = 10,000 ms, BW = 1050 Hz/px, 86 contiguous slices with an acquisition matrix of 170 × 170, voxel size = 1.2 mm × 1.2 mm × 1.2 mm, and TA = 14:20 min:s. To reduce geometric distortions partial parallel under sampling [GRAPPA (Griswold et al., 2002)] with an acceleration factor of 3 and 45 reference lines was applied in the phase-encoding direction.

In addition, a field-map was acquired with a 2D double-echo gradient-echo sequence to correct residual geometric distortions of the diffusion-weighted images caused by susceptibility differences between air-bone and air-tissue interfaces. The field-map was recorded with TE_1_ = 6 ms, TE_2_ = 7 ms, TR = 1000 ms, FA = 50°, 51 contiguous slices, matrix = 102 × 102, voxel size = 2 mm × 2 mm × 2 mm, and TA = 3:22 min:s.

### Data processing

#### Relaxometry

Maps of the longitudinal relaxation times were calculated directly at the MRI scanner by applying the MP2RAGE reconstruction framework provided by the manufacturer. The relaxation times were subsequently converted to relaxation rates according to the relation *R*_1_ = 1/*T*_1_ to facilitate better depiction of anatomical structures.

Maps of the effective transverse relaxation rate, $R_{2}^{*}$, were obtained using the power method (McGibney and Smith, 1993; Miller and Joseph, 1993), i.e., the squared magnitude signal decay, *S*(r→, *TE*)^2^, of the 3D multi-echo GRE scan was used for the regression (McGibney and Smith, 1993; Miller and Joseph, 1993):

(1) $$S{\left(\vec{r},\;TE\right)}^{2}=S_{0}{\left(\vec{r}\right)}^{2}\cdot \exp\left(-2\cdot TE\cdot R_{2}^{*}\left(\overset{\to }{r}\right)\right),$$

where $S_{0}\left(\vec{r}\right)$ is the signal intensity at *TE* = 0 and $\vec{r}$ is the position vector. This approach reduces contamination of Rician noise to the fit and provides more accurate relaxation rates than fitting a mono-exponential model to the magnitude signal decay $S(\vec{r},\text{ TE})$ (van der Weerd et al., 2000).

#### Quantitative susceptibility mapping

Single-channel GRE magnitude images were combined using the sum-of-squares method (Roemer et al., 1990), whereas single-channel GRE phase images were combined by taking into account the channel-dependent phase offset, which was estimated from the single-channel images at different echo times by voxel-wise linear fitting of the phase evolution (Robinson et al., 2011). Phase aliasing in the combined GRE phase data was resolved using a 3D Laplacian-based phase unwrapping algorithm (Schofield and Zhu, 2003) and phase images of different echo times were then combined in a CNR-optimized manner according to (Wu et al., 2012). Background phase contributions were eliminated with sophisticated harmonic artifact reduction for phase data (SHARP) (Schweser et al., 2011) (regularization parameter: 0.05) combining 10 different spherical kernels with varying radii ranging from 0.43 to 4.3 mm (Li et al., 2011). Susceptibility mapping was performed using homogeneity enabled incremental dipole inversion (HEIDI), which incorporates *a priori* information extracted from the complex GRE signal to address the ill-posed nature of QSM (Schweser et al., 2012). Since the calculated susceptibility values represent relative rather than absolute values (Schweser et al., 2011), susceptibility differences were specified with respect to a homogenous region of normal appearing white matter (NAWM, semioval center).

#### Track-density imaging

Geometric distortion of diffusion-weighted images was corrected using FUGUE (FSL toolbox, FMRIB, Oxford, England) based on the additionally acquired field-map. The unwarped DWI data were then processed using the MRtrix software package (Brain Research Institute, Melbourne, VIC, Australia) to produce track-density images as proposed in Calamante et al. (2010). Processing steps performed with MRtrix included the calculation of tensor data as well as fractional anisotropy maps, estimation of the response function, spherical deconvolution, probabilistic tractography and track-density mapping. Only voxels with fractional anisotropy, derived from the tensor model, that exceeded a threshold of 0.7 were taken into account for estimating the coefficients of the response function based on DWI data (Tournier et al., 2004). These coefficients and a maximum harmonic order of eight were used for constrained spherical deconvolution of DWI data (Tournier et al., 2007). This approach allowed modeling of multiple fiber populations within an imaging voxel, thereby overcoming the well-known limitation of the diffusion tensor model in regions with crossing fibers. Probabilistic streamline tracking was carried out using the second order integration over fiber orientation distributions (iFOD2) algorithm (Tournier et al., 2010) by seeding randomly throughout the predefined mask covering the whole brainstem and using the following relevant parameters: step size = 0.1 mm, number of fibers = 800,000, minimum curvature radius = 0.4 mm and a maximum number of 2000 trials for each point. In order to generate the track-density images, a virtual regularly spaced grid was superposed with a resolution of 0.43 mm × 0.43 mm × 0.43 mm on the tractography results and the total number of fiber tracks present in each grid element was calculated. Hereby, the super-resolution property is achieved by utilizing the additional information provided by modeling the fiber tracking results (Calamante et al., 2010). The track-density images were calculated on a virtual isotropic resolution of 0.43 mm to facilitate comparison with the $R_{2}^{*}$ and susceptibility maps. Finally, the track-density images were direction-encoded by assigning each grid element an RGB color that represents the local fiber orientation as given by averaging the colors of all the fiber tracking (streamline) segments contained within each grid element.

### Data analysis

All MRI contrasts were converted into an identical space for visual inspection. To this end, the *R*_1_ maps, track-density images and *T*_2_-weighted images were linearly transformed into the space of the susceptibility maps based on orientation information encoded in the DICOM images. The susceptibility maps were chosen as target to maintain the high spatial resolution of the susceptibility and $R_{2}^{*}$ maps. Manual reformatting of quantitative susceptibility maps in multi-planar orientations was performed with Freeview of the Freesurfer software library^1^ and the resulting rigid transformation matrix was applied to the previously registered MR image contrasts and to the $R_{2}^{*}$ maps. The MR images were averaged across three adjacent slices for signal-to-noise improvement, resulting in an effective slice thickness of 1.3 mm.

For qualitative comparison horizontal histological sections obtained from a 36-year-old male stained for myelin and for cells were consulted. These histological images were courtesy of the Brain Biodiversity Bank of the Michigan State University^2^^,^^3^ with support from the US National Science Foundation. An experienced neuroanatomist (UB; experience more than 15 years) assessed the MR images and identified anatomical structures and substructures if they coincided with a histoarchitectonic atlas (Bergman et al., 1989; Naidich et al., 2009; Paxinos et al., 2012) and if they were identifiable with respect to the surrounding tissue. For this qualitative analysis the window and level settings of the MR images were adjusted freely.

For quantitative characterization the contrast-to-noise ratio (CNR) of selected brain structures [red nucleus, substantia nigra, central tegmental tract, superior colliculus, inferior colliculus, reticulotegmental nucleus, middle cerebellar peduncle (MCP), superior cerebellar peduncle (SCP), and transverse pontine fibers] was determined across the different MR image contrasts (*R*_1_, $R_{2}^{*}$, susceptibility, and *T*_2_-weighted contrast) according to:

(2) $$\mathrm{CN}R_{\mathrm{cy}}=\left|\frac{S_{\mathrm{cy}}-S_{\mathrm{cys}}}{\sigma }\right|\cdot \eta_{c}.$$

CNR_cy_ denotes the CNR for MR image contrast *c* and anatomic region *y*. Mean image intensities measured in a volume-of-interest (VOI) of the investigated structure *y* and its surrounding tissue are denoted by *S*_cy_ and *S*_cys_, respectively. The standard deviation of the signal intensities measured in a VOI of normal appearing white matter (semioval center) was used as an estimate of noise, σ. To account for variations in spatial resolution of the different investigated contrasts, the CNR was normalized to a voxel volume of 1 mm × 1 mm × 1 mm as suggested in (Nölte et al., 2012). To this end, the normalization factor η_c_ is introduced in Eq. 2, which is defined as the ratio between 1 mm^3^ and the acquired voxel volume of image contrast *c*. CNR was not determined on diffusion-weighted data because of their incompletely compensated geometric distortions.

To calculate the CNR and to measure both the relaxation rates and magnetic susceptibility, VOIs were identified based on magnetic susceptibility, *R*_1_, and $R_{2}^{*}$ maps in the coordinate space of the susceptibility maps for each subject in both hemispheres. The VOIs were then transferred to the space of the *R*_1_ maps and the *T*_2_-weighted data, respectively, by employing orientation information encoded in the DICOM images and nearest-neighbor interpolation. Mean values and standard deviation of CNR and of the quantitative tissue parameters (*R*_1_, $R_{2}^{*}$, and magnetic susceptibility) were then computed across hemispheres and subjects.

## Results

All qualitative findings presented in this study were consistent across all subjects unless otherwise specified.

**Figure 1:**
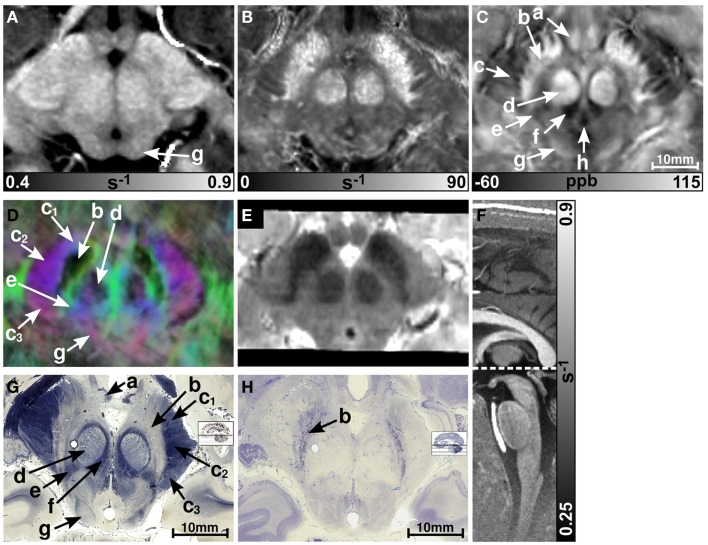
**Images of the midbrain**. *R*_1_, $R_{2}^{*}$, and susceptibility maps as well as direction-encoded track-density and *T*_2_-weighted images of the same brain region are presented in axial orientation from **(A–E)**, respectively. Red, blue, and green in the track-density image represent anisotropy along medial-lateral, superior-inferior, and anterior-posterior directions, respectively. Note that the track-density image is slightly distorted compared to the other image contrasts **(A–C,E)**. The sectional plane of the axial images is indicated by the dashed line overlaid on the sagittal *R*_1_ map shown in **(F)**. Axial histological sections from a different subject stained for myelin and cells are illustrated in **(G)** and **(H)**, respectively. The arrows in the images indicate: (a) mammillary body, (b) substantia nigra, (c) crus cerebri, (c_1_) corticobulbar fibers, (c_2_) corticospinal fibers, (c_3_) corticopontine fibers, (d) red nucleus, (e) medial lemniscus, (f) central tegmental tract, (g) inferior colliculus, and (h) medial longitudinal fasciculus. [The histological stains **(G,H)** were adapted with permission from http://www.brains.rad.msu.edu and http://brainmuseum.org, supported by the US National Science Foundation.]

**Figure 2:**
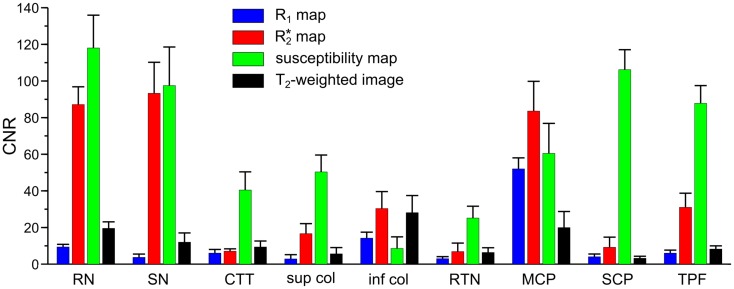
**Contrast-to-noise ratio (CNR) of selected anatomical regions**. Mean values of CNR across six subjects and both hemispheres were extracted from *R*_1_ (blue bars), $R_{2}^{*}$ (red bars), and susceptibility maps (green bars) as well as *T*_2_-weighted images (black bars). Brain regions include: RN - red nucleus vs. surrounding tissue, SN - substantia nigra vs. tissue between red nucleus and substantia nigra, CTT - central tegmental tract vs. surrounding tissue (without red nucleus), sup col - superior colliculus vs. adjacent tissue, inf col - inferior colliculus vs. adjacent tissue, RTN - reticulotegmental nucleus vs. surrounding white matter tissue, MCP - middle cerebellar peduncle vs. adjacent cerebrospinal fluid, SCP - superior cerebellar peduncle vs. reticular formation, TPF - transverse pontine fibers vs. corticospinal tract. The error bars indicate the 95%-confidence interval.

Figure 1 presents representative MR images and corresponding histological sections of the midbrain. The red nuclei and the substantia nigra were most strikingly discernible on the $R_{2}^{*}$ (Figure 1B) and susceptibility maps (Figure 1C) as well as on the direction-encoded track-density (Figure 1D) and *T*_2_-weighted images (Figure 1E), but were barely visible on the *R*_1_ map (Figure 1A). This qualitative finding was also supported by the CNR measurements presented in Figure 2. On the track-density image (Figure 1D) both nuclei showed lower anisotropy as reflected in the reduced color saturation (arrows b and d). Different fiber bundles traversing the crus cerebri [corticobulbar fibers (arrow c_1_), corticospinal fibers (arrow c_2_), and corticopontine fibers (arrow c_3_)] could be identified on the track-density images. In the midbrain, interestingly, the central tegmental tract (arrow f) and the medial lemniscus (arrow e, five of six subjects) were only identifiable reliably on susceptibility maps, whereas the mammillary body (Figure 1; arrow a) was discernible on all MR image contrasts except the track-density images. The superior (not shown) and inferior colliculi (Figure 1; arrow g) were distinguishable across all subjects only on the *R*_1_ maps. Both of these structures could be assessed on the other MRI contrasts in at least four of six subjects (superior colliculus on susceptibility maps in six of six subjects). The superior colliculus and inferior colliculus presented highest CNR on susceptibility maps and $R_{2}^{*}$ maps, respectively (Figure 2). Anatomic regions with low magnetic susceptibility (Figure 1C; arrows c, e, f), such as the hypointense rim around the red nuclei coincided with regions of high myelin content (Figure 1G; arrows c_1–3_, e, f) and had no unique imaging correlate on any of the other MR images. The histological stains (Figures 1G,H) showed increased myelin and decreased cell density in the red nucleus compared to the substantia nigra.

**Figure 3:**
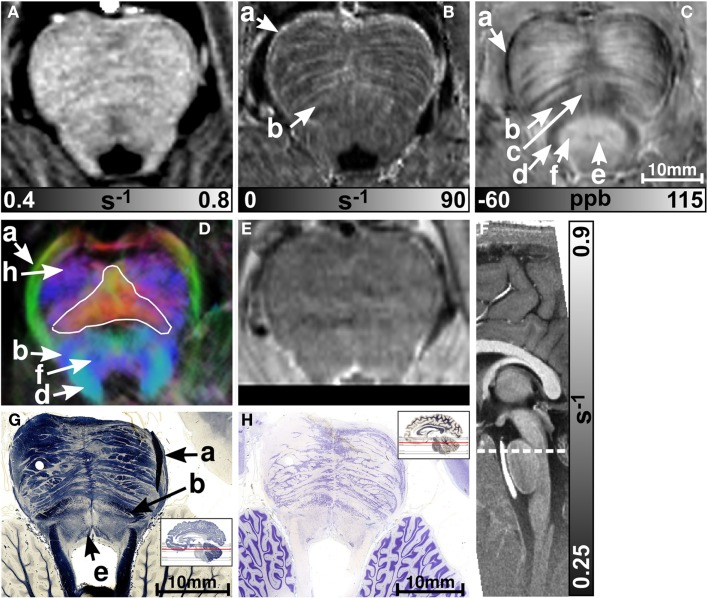
**Images of the rostral part of the pons**. *R*_1_, $R_{2}^{*}$, and susceptibility maps as well as direction-encoded track-density (same color-encoding as in Figure 1; slightly distorted compared to the other axial image contrasts) and *T*_2_-weighted images of the same region are presented in axial orientation from **(A–E)**, respectively. The sectional plane of the axial images is indicated by the dashed line overlaid on the sagittal *R*_1_ map shown in **(F)**. Axial histological sections from a different subject stained for myelin and cells are illustrated in **(G)** and **(H)**, respectively. The arrows in the images indicate: (a) middle cerebellar peduncle, (b) medial lemniscus, (c) reticulotegmental nucleus, (d) superior cerebellar peduncle, (e) medial longitudinal fasciculus, (f) central tegmental tract, and (h) corticospinal tract. The solid white outline in **(D)** indicates the pontocerebellar fibers. [The histological stains **(G,H)** were adapted with permission from http://www.brains.rad.msu.edu and http://brainmuseum.org, supported by the US National Science Foundation.]

**Figure 4:**
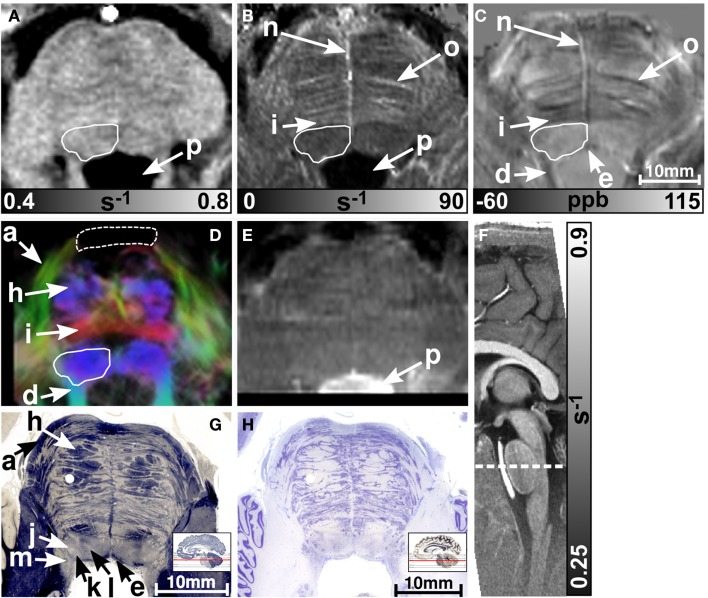
**Images of the middle part of the pons**. *R*_1_, $R_{2}^{*}$, and susceptibility maps as well as track-density (same color-encoding as in Figure 1; slightly distorted compared to the other axial image contrasts) and *T*_2_-weighted images of the same region are presented in axial orientation from **(A–E)**, respectively. The sectional plane of the axial images is indicated by the dashed line overlaid on the sagittal *R*_1_ map shown in **(F)**. Axial histological sections from a different subject stained for myelin and cells are illustrated in **(G)** and **(H)**, respectively. The arrows in the images indicate: (a) middle cerebellar peduncle, (d) superior cerebellar peduncle, (e) medial longitudinal fasciculus, (h) corticospinal fibers, (i) pontocerebellar tract, (j) facial nucleus, (k) salivatory nucleus, (l) abducens nucleus, (m) solitary nucleus, (n) vein, (o) fiber bundle that traverses the pons in medial to lateral direction, and (p) fourth ventricle (cerebrospinal fluid). The solid white outline in **(A–D)** summarizes the caudal part of the pontine reticular nucleus, the facial nucleus, salivatory nucleus, and abducens nucleus. The dashed white outline in **(D)** indicates a region between the middle cerebellar peduncle that could not be resolved with TDI. [The histological stains **(G,H)** were adapted with permission from http://www.brains.rad.msu.edu and http://brainmuseum.org, supported by the US National Science Foundation.]

MR images of the rostral and the middle part of the pons are depicted in Figures 3 and 4, respectively. The $R_{2}^{*}$ and magnetic susceptibility maps clearly displayed fibers traversing the pons in medial-lateral direction and fibers of the middle cerebellar peduncle (MCP) (arrow a in Figure 3) as also indicated by the myelin stains (Figures 3G and 4G). These fiber structures were barely visible on the *R*_1_ maps [Figures 2 (TPF), 3A, and 4A]. The direction of the transverse pontine fibers could not be reliably separated from the superior-inferior running corticospinal tract on the track-density images (Figures 3D and 4D). In contrast, the susceptibility maps revealed even smaller fiber tracts [e.g., central tegmental tract (arrow f, two of six subjects), medial longitudinal fasciculus (arrow e)] and nerve nuclei [reticulotegmental nucleus (arrow c)] that were indiscernible on *R*_1_ and $R_{2}^{*}$ maps as well as on the *T*_2_-weighted images. In the direction-encoded track-density images larger [e.g., MCP (arrow a), superior cerebellar peduncle (arrow d), corticospinal tract (arrow h), pontocerebellar fibers (white solid outline in Figure 3D)] and smaller [e.g., central tegmental tract (arrow f)] fiber tracts were clearly identifiable. The MCP was also discernible on the *R*_1_, $R_{2}^{*}$ and susceptibility maps, whereas the superior cerebellar peduncle displayed on the *R*_1_ and the susceptibility maps on all subjects and in two of six subjects on the $R_{2}^{*}$ maps. Both of these structures (MCP, SCP) were also barely visible on *T*_2_-weighted images (MCP in three subjects, SCP in one subject; Figure 2). The susceptibility maps showed the highest CNR for nearly all investigated structures within the pons (Figure 2). Only the CNR value of MCP with respect to adjacent cerebrospinal fluid were outperformed by the $R_{2}^{*}$ maps. Although pontine veins were only seen on both $R_{2}^{*}$ and susceptibility maps (Figures 4B,C; arrow n), their identification compared to adjacent tissue structures (e.g., transverse pontine fibers) was substantially improved on susceptibility maps. The solid white outline in Figures 4A–D encloses the caudal part of the pontine reticular nucleus, the facial nucleus, salivatory nucleus and nucleus abducens. Although *R*_1_, $R_{2}^{*}$, and susceptibility maps displayed heterogeneous signal intensities in this area, discrimination of these nuclei was not possible. The interruption of the fiber tracts between the MCP seen on the track-density image (dashed outline in Figure 4D) is most likely caused by data acquisition and TDI post-processing issues (incompletely corrected geometric distortions in the unwarped diffusion-weighted images that impeded reliable TDI post-processing) because no such interruption is observed on the susceptibility map (Figure 4C) and on the histological stains (Figures 4G,H). The *T*_2_-weighted images (Figures 3E and 4E) displayed rather homogeneously with low contrast (Figures 2–4), which prevented reliable identification of any substructures across all subjects.

**Figure 5:**
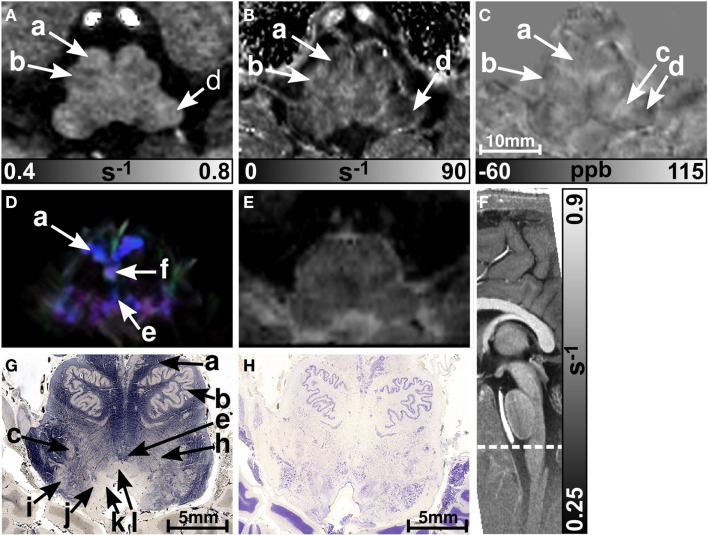
**Images of the rostral part of the medulla oblongata**. *R*_1_, $R_{2}^{*}$, and susceptibility maps as well as direction-encoded track-density (same color-encoding as in Figure 1; slightly distorted compared to the other axial image contrasts) and *T*_2_-weighted images of the same region are presented in axial orientation from **(A–E)**, respectively. The sectional plane of the axial images is indicated by the dashed line overlaid on the sagittal *R*_1_ map shown in **(F)**. Axial histological sections from a different subject stained for myelin and cells are illustrated in **(G)** and **(H)**, respectively. The arrows indicate: (a) pyramid, (b) inferior olive, (c) spinal trigeminal nucleus, (d) inferior cerebellar peduncle, (e) medial longitudinal fasciculus, (f) medial lemniscus, (h) reticular formation, (i) accessory cuneate nucleus, (j) solitary nucleus, (k) dorsal motor nucleus of the vagus nerve, and (l) hypoglossal nucleus. [The histological stains **(G,H)** were adapted with permission from http://www.brains.rad.msu.edu and http://brainmuseum.org, supported by the US National Science Foundation.]

**Figure 6:**
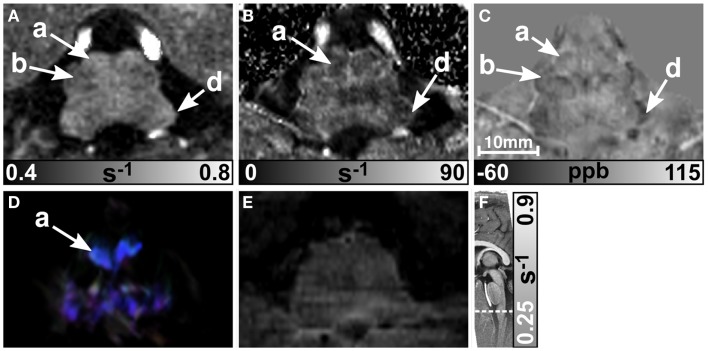
**Images of the middle part of the medulla oblongata**. *R*_1_, $R_{2}^{*}$, and susceptibility maps as well as track-density (same color-encoding as in Figure 1; slightly distorted compared to the other axial image contrasts) and *T*_2_-weighted images of the same region are presented in axial orientation from **(A–E)**, respectively. The sectional plane of the axial images is indicated by the dashed line overlaid on the sagittal *R*_1_ map shown in **(F)**. The arrows in the images indicate: (a) pyramid, (b) inferior olive, and (d) inferior cerebellar peduncle.

Figures 5 and 6 display the rostral and middle part of the medulla oblongata, respectively. The pyramid (arrow a) is located anterior to the inferior olive and was clearly visible on all contrasts (four of six subjects on $R_{2}^{*}$ maps) except the *T*_2_-weighted images. It could be well discriminated from the inferior olive (arrow b) which was also clearly discernible on the *R*_1_ (five subjects) and susceptibility map (four subjects), and less obvious on $R_{2}^{*}$ maps (three subjects). The inferior cerebellar peduncle was clearly visible on track-density images and was distinguishable on *R*_1_, $R_{2}^{*}$, and susceptibility maps in four subjects at least. Only the susceptibility maps enabled identification of the spinal trigeminal nucleus (arrow c in Figure 5; four subjects). Although the histological stains (Figures 5G,H) suggested the presence of several cranial nuclei, further nuclei were not visible on the MR images.

Finally, *R*_1_, $R_{2}^{*}$, and susceptibility differences are listed in Table 1 for selected anatomical regions. The differences of the susceptibility and the $R_{2}^{*}$ values between fibers running nearly parallel (transverse pontine fibers) and nearly perpendicular (corticospinal tract) to the static magnetic field were (−49 ± 11) ppb and (8.9 ± 6) s^−1^, respectively.

**Table 1:** Mean values of longitudinal relaxation rate (*R*_1_), effective transverse relaxation rate ($R_{2}^{*}$), and volume magnetic susceptibility difference (Δχ) with respect to normal appearing white matter over six subjects and both brain hemispheres for selected anatomical regions.

Region	Structure	*R*_1_ (s^−1^)	$R_{2}^{*}$(s^−1^)	Δχ (ppb)
Midbrain	Red nucleus	0.809 ± 0.029	61.3 ± 6	84 ± 8
	Substantia nigra	0.759 ± 0.031	69.4 ± 3	82 ± 13
	Crus cerebri	0.777 ± 0.029	29.8 ± 3	−27 ± 18
Pons	Reticulotegmental nucleus	0.704 ± 0.043	34.5 ± 3	−6 ± 12
	Corticospinal tract	0.684 ± 0.042	27.1 ± 4	25 ± 19
	Transverse pontine fibers	0.723 ± 0.036	36.0 ± 5	−24 ± 19
	Pontocerebellar tract	0.693 ± 0.033	38.3 ± 2	−26 ± 16
CSF	Cerebrospinal fluid	0.300 ± 0.006	0.9 ± 0.4	−6 ± 11

*The values are presented as mean ± standard deviation*.

## Discussion

We have investigated the anatomy of the human brainstem *in vivo* by applying MR imaging at 7 T with high spatial, isotropic resolution, and using multiple image contrasts. QSM and TDI have been applied jointly for the first time to identify anatomical substructures of the brainstem. Both MR methods directly reflect subtle variations in tissue composition that were found to be consistent with histology as demonstrated with corresponding histological stains selected from a data base. In contrast, longitudinal relaxation rate mapping and *T*_2_-weighted imaging both performed inferiorly in their ability to delineate anatomical details (Figure 2).

$R_{2}^{*}$ relaxation comprises both intrinsic microscopic transverse relaxation (R_2_) and relaxation due to heterogeneous magnetic susceptibility distributions on a mesoscopic or macroscopic scale $\left(R_{2}^{\prime }\right)$. In contrast to the $R_{2}^{*}$ relaxation rate, which increases with the concentration of both iron (Langkammer et al., 2010) and myelin (Lee et al., 2012), magnetic susceptibility shows a different dependence on the concentration of these substances, i.e., higher magnetic susceptibility values with increasing iron concentration (Langkammer et al., 2012b), and lower magnetic susceptibility values with increasing myelin density (Liu et al., 2011; Schweser et al., 2011; Li et al., 2012a,b). Hence, image contrast on $R_{2}^{*}$ and susceptibility maps appears complementary. The longitudinal relaxation rate, *R*_1_, in turn is heavily influenced by myelin content, but shows little effect with respect to tissue iron concentration (Bridge and Clare, 2006).

Both $R_{2}^{*}$ maps and susceptibility maps provided detailed depiction of the non-cranial nuclei and nerve fibers in the midbrain in accordance with recent studies (Schweser et al., 2011; Deistung et al., 2013a). In particular, the red nuclei and the substantia nigra were clearly discernible on susceptibility and $R_{2}^{*}$ maps because both structures contain substantially higher iron concentrations compared to the surrounding brainstem tissue (Morris et al., 1992). Interestingly, the highly myelinated rim around the red nuclei, which was clearly seen on the myelin stain and on the susceptibility map was not visible on the *R*_1_ map, potentially due to the lower spatial resolution of the MP2RAGE acquisition compared to the gradient-echo acquisition.

Brainstem nuclei were barely seen on the *R*_1_, $R_{2}^{*}$ and susceptibility maps sectioning the pons and medulla oblongata; however, the inferior olive (Figures 5 and 6, arrow b) could be identified and both the spinal trigeminal nucleus (Figure 5C, arrow c) and the reticulotegmental nucleus (Figure 3C, arrow c) were discernible on the susceptibility maps. The inferior olive is the sole source of the climbing fibers to the Purkinje cells in the cerebellar cortex and is involved in learning and timing of motor behavior (De Zeeuw et al., 1998). Lesions in the inferior olive can thus introduce restrictions in performing specialized motor tasks (Martin et al., 1996). The spinal trigeminal nucleus is the largest of all cranial nerve nuclei and one of the three paired sensory nuclei associated with the trigeminal nerve. It receives input from pain, temperature, and some tactile afferents in the trigeminal nerve and is involved in migraine headache (Kaube et al., 2002). The reticulotegmental nucleus of the pons is an important component of the oculomotor circuit that regulates horizontal saccades and smooth pursuit movements of the eyes (Keller and Crandall, 1981; Büttner-Ennever and Horn, 1997). Damage to the reticulotegmental nucleus has been observed in spinocerebellar ataxia (Rüb et al., 2004) and it most probably contributes to hypometric horizontal saccades and slowing of smooth pursuits that characteristically develop in patients suffering from Alzheimer’s disease (Rüb et al., 2001). Therefore, non-invasive assessment of these nuclei may help to improve diagnosis and understanding of these diseases.

These nuclei (inferior olive, spinal trigeminal nucleus, reticulotegmental nucleus) were visible on the susceptibility maps because of their different iron concentration and/or myelin content with respect to surrounding tissue. A histological study by Morris et al. (1992), for instance, demonstrated iron accumulation in the nuclei of the dorsal parts of the brainstem, the pyramids, the spinal trigeminal nucleus, and the inferior olive (although with substantially lower iron concentration than in the red nuclei and substantia nigra) and reported only little or no intrinsic iron reactivity for many other nuclei and tracts of the brainstem (e.g., solitary nucleus, hypoglossal nucleus, raphe pallidus nucleus, cuneate fasciculus, and gracile fasciculus). Brainstem nuclei have a similar, relatively low myelin density as seen from the histological myelin stains. Only the inferior olive (Figure 5G, arrow b) and the spinal trigeminal nucleus (Figure 5G, arrow c) and reticulotegmental nucleus [see (Paxinos et al., 2012) and Figure 3C, arrow c] are embedded in tissue with substantially increased myelin density.

In addition to myelin’s bulk diamagnetic nature, a dependence of the magnetic susceptibility on the axons’ alignment with respect to the external magnetic field has been recently reported (Liu, 2010; Li et al., 2012a; Schweser et al., 2012; Wharton and Bowtell, 2012). Similar direction dependent observations have been made for $R_{2}^{*}$ (Bender and Klose, 2010; Denk et al., 2011; Lee et al., 2011; Sati et al., 2012). We measured differences of the susceptibility and the $R_{2}^{*}$ values between fibers running nearly parallel (transverse pontine fibers) and nearly perpendicular (corticospinal tract) to the static magnetic field of (−49 ± 11) ppb and (8.9 ± 6) s^−1^, respectively. This susceptibility difference is nearly threefold as large as the values that have been derived for a hollow cylinder model of myelin (−18 to −16 ppb) (Li et al., 2012a; Wharton and Bowtell, 2012). *In vivo*, however, Li et al., 2012a also reported susceptibility differences between parallel and perpendicular fibers of up to −40 ppb in individual white matter regions, a value which is almost the same as reported here. The result for the orientation dependence of $R_{2}^{*}$ is in good agreement with the recently reported *ex vivo* value of −6.44 ± 0.15 s^−1^ at 7 T (Lee et al., 2011). The orientation-dependent differences of magnetic susceptibility and $R_{2}^{*}$in our study may be caused by the presence of additional contributors other than myelin, such as pontine nuclei and neuropil within the investigated volumes-of-interest. Nevertheless, consistent with the theory of myelin’s orientation dependency, the contrast between fibers running in medial-lateral direction (oriented nearly perpendicular to the magnetic field; e.g., transverse pontine fibers, arrow o in Figure 4) and fibers running inferior-superiorly (oriented nearly parallel to the magnetic field; corticospinal tract) is enhanced on the $R_{2}^{*}$ and susceptibility maps. More sophisticated approaches, such as susceptibility tensor imaging (STI) (Liu, 2010; Li et al., 2012a) or multipole susceptibility tensor imaging (MSTI) (Liu and Li, 2013) may further improve discrimination between fibers and nuclei of the pons and medulla, particularly in combination with high spatial resolution.

Direction-encoded TDI enabled discrimination of the directionality of nerve fibers within the brainstem and yielded complementary information to the relaxation and susceptibility maps. The depiction of larger fiber bundles was consistent with the results of other DTI studies conducted *in vivo* at lower magnetic fields (Nagae-Poetscher et al., 2004; Salamon et al., 2005). We were, however, unable to resolve unambiguously the transverse pontine fibers, which were clearly visible on the $R_{2}^{*}$ and susceptibility maps (Figure 4). This may be due to the lower base voxel resolution of 1.2 mm of the DWI data, which was chosen as a compromise between spatial resolution, SNR, and acquisition time. To overcome this limitation we interpolated the DWI data using a super-resolution approach (TDI) to obtain an isotropic virtual resolution of 0.43 mm. It should be noted, however, that the TDI technique is not able to fully recover the whole information that would be present if the data would be acquired with such a high spatial resolution (Calamante et al., 2011). Due to the larger errors in tractography by performing fiber tracking on data with a lower resolution, the higher resolved track-density images appeared slightly blurred and lost some fine details; explaining the difficulties in identifying the transverse pontine fibers in the presented track-density images (Figures 3D and 4D). As might be expected, the level of blurring in the track-density images depends on the desired virtual resolution (Calamante et al., 2011). Higher spatial resolution is thus needed to delineate transverse pontine fibers unambiguously, which requires sophisticated modifications of the diffusion MRI sequence such as combining zoomed imaging and parallel imaging (Heidemann et al., 2012; Eichner et al., 2013). Karampinos et al. (2009) were recently able to resolve transverse pontine fibers in direction-encoded fractional anisotropy maps with a resolution of 0.8 mm × 0.8 mm × 3 mm at 3T by applying a dedicated acquisition scheme (self-navigated, multi-shot, variable-density, spiral-imaging with outer volume suppression).

One particular problem of brainstem imaging with MRI are the induced field inhomogeneities caused by nearby air-tissue or bone-tissue interfaces. In the vicinity of such interfaces significant image distortions of EPI-based diffusion-weighted images occur and additional spin dephasing arises in gradient-echo images. Despite unwarping the diffusion-weighted images based on gradient-echo field-map information, it was not possible to completely compensate geometric distortions, thus impeding accurate depiction of ventral fiber structures of the middle pons and medulla oblongata on track-density images. These induced field inhomogeneities manifest themselves as steep gradients in gradient-echo phase images and lead to signal reductions in gradient-echo magnitude images and decreased SNRs, affecting both susceptibility maps [due to increased phase noise (Gudbjartsson and Patz, 1995)] and $R_{2}^{*}$ maps. In future studies, the impact of spin dephasing due to air-tissue or bone-tissue interfaces on $R_{2}^{*}$ maps may be minimized by taking into account the macroscopic field inhomogeneity reflected in the phase images (de Leeuw and Bakker, 2012). *R*_1_ maps and *T*_2_-weighted images, on the other hand, are distinctly more immune against these field inhomogeneity effects.

The quantitative analysis revealed similar values for the longitudinal relaxation rates (*R*_1_) for nuclei and myelinated fibers (Table 1), whereas susceptibility and $R_{2}^{*}$ values varied more substantially. This broader spread of $R_{2}^{*}$ and susceptibility values is reflected in the large CNR values (Figure 2) underlining the good discrimination of nerve fibers on susceptibility maps and the exquisite delineation of midbrain nuclei on susceptibility and $R_{2}^{*}$ maps. It should, however, be noted that the discrimination of brain tissue with respect to cerebral spinal fluid (CSF) on susceptibility maps is not as striking as on the *R*_1_, $R_{2}^{*}$, and *T*_2_-weighted images [Table 1; Figure 4 (arrow p)]. Due to the quantitative nature of *R*_1_, $R_{2}^{*}$, and magnetic susceptibility maps, these contrasts may be combined by projections along support vectors observed from discriminant analysis or support vector machines to generate composite images with improved depiction of anatomical features while providing improved discrimination of CSF (Deistung et al., 2013b).

In conclusion, maps of magnetic susceptibility displayed the largest number of brainstem structures, including larger and smaller fiber pathways as well as several nerve nuclei. Usage of multiple image contrasts enables a detailed non-invasive view into tissue structure and composition. Hence, multi-contrast MR imaging of the brainstem at ultra-high magnetic fields that utilizes relaxation, diffusion, and magnetic susceptibility information is a versatile tool to assess anatomy individually in great detail.

## Conflict of Interest Statement

The authors declare that the research was conducted in the absence of any commercial or financial relationships that could be construed as a potential conflict of interest.

## Footnotes

^1^http://surfer.nmr.mgh.harvard.edu/

^2^http://www.brains.rad.msu.edu

^3^http://brainmuseum.org

## Abbreviations

DTI, diffusion tensor imaging; GRE, gradient recalled echo; QSM, quantitative susceptibility mapping; ppb, parts per billion; $R_{2}^{*}$, effective transverse relaxation rate; SHARP, sophisticated harmonic artifact reduction for phase data; TDI, track-density imaging.
